# Patterned growth of carbon nanotubes over vertically aligned silicon nanowire bundles for achieving uniform field emission

**DOI:** 10.1186/1556-276X-9-540

**Published:** 2014-10-01

**Authors:** Yung-Jr Hung, Yung-Jui Huang, Hsuan-Chen Chang, Kuei-Yi Lee, San-Liang Lee

**Affiliations:** 1 Department of Photonics, National Sun Yat-sen University, No. 70, Lienhai Rd., Kaohsiung 80424, Taiwan; 2 Department of Electronic Engineering, National Taiwan University of Science and Technology, No. 43, Sec. 4, Keelung Rd., Taipei 106, Taiwan

**Keywords:** Silicon nanowire, Carbon nanotube, Metal-induced chemical etching, Field emission, Electrostatic screening effect

## Abstract

A fabrication strategy is proposed to enable precise coverage of as-grown carbon nanotube (CNT) mats atop vertically aligned silicon nanowire (VA-SiNW) bundles in order to realize a uniform bundle array of CNT-SiNW heterojunctions over a large sample area. No obvious electrical degradation of as-fabricated SiNWs is observed according to the measured current-voltage characteristic of a two-terminal single-nanowire device. Bundle arrangement of CNT-SiNW heterojunctions is optimized to relax the electrostatic screening effect and to maximize the field enhancement factor. As a result, superior field emission performance and relatively stable emission current over 12 h is obtained. A bright and uniform fluorescent radiation is observed from CNT-SiNW-based field emitters regardless of its bundle periodicity, verifying the existence of high-density and efficient field emitters on the proposed CNT-SiNW bundle arrays.

## Background

One-dimensional nanomaterials have attracted a tremendous amount of attention in recent years due to their interesting electronic and optical properties and a diverse array of potential for nanoscale device applications [1,2]. Among the promising nanoelectronic applications, field emission and related devices have become the subject of intense fundamental and technological investigations because of their great potential for industrial applications, in particular as flat panel displays and electron guns [3-10]. Reports had shown that carbon nanotube (CNT)-based field emitter exhibits excellent field emission properties due to its superior thermal and electrical characteristic [11]. However, the wire number density of as-grown CNTs is usually as high as 10^10^ CNTs/cm^2^[12]. Such a dense CNT structure would suffer from the so-called electrostatic screening effect provoked by the proximity of neighboring wires which results in limited field emission performance. Alternatively, a pillar array of aligned CNT bundles had shown better field emission performance due to the release of the electrostatic screening effect [13]. However, it is difficult to maintain the verticality of CNT bundles as the bundle periodicity becomes submicron scale to maximize the number of effective field emitters. Therefore, instead of using CNTs as both field emitter material and the supporting structure to obtain certain aspect ratio in conventional CNT bundle arrays, this work proposes to use vertically aligned silicon nanowires (VA-SiNWs) as the supporting template structures, while CNTs only serve as the emitter material. Recent results also focused on the synthesis of hybrid CNT-SiNW heterojunction array in order to take the advantages from both material systems for breakthrough findings in not only high performance electron field emitters but also many other emerging technologies [14-16]. We had previously demonstrated that VA-SiNWs could be realized with top-down wet chemical etching using a thin silver film as the catalyst [17]. We also developed several fabrication processes to realize an array of VA-SiNW bundles using thin chemical oxide or thick photoresist (PR) as the mask to define the arrayed structure [18,19]. In this work, mesa-type arrays of VA-SiNW bundles are realized to serve as an arrayed template for the growth of coarse CNT mats atop their surfaces. The proposed hybrid device leverages from the metallic-like property of CNTs and mature and flexible processing technologies available in silicon. The bundle arrangement of SiNW templates is optimized in order to release the electrostatic screening effect and to maximize the field enhancement factor. The material and electrical characterization of as-realized CNT-SiNW field emitter array as well as its emission uniformity and stability tests are conducted and discussed.

## Methods

### Synthesis of CNT-SiNW heterojunction bundle array

**Figure 1:**
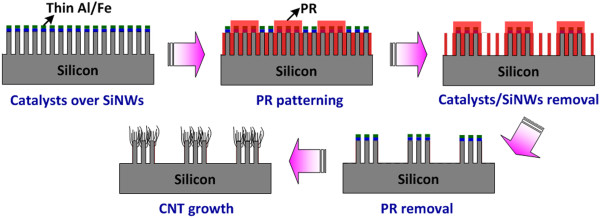
Preparation procedures of CNT-SiNW bundle arrays.

The fabrication procedure to realize the CNT-SiNW heterojunction bundle array is shown in Figure 1. The synthesis of VA-SiNW arrays is carried out on front-side polished, boron-doped (100) crystalline silicon wafers with an improved metal-induced chemical etching method which utilizes a thin silver film as catalysts to avoid the agglomeration issue of nanowires. As the facts that the motion of silver particles is preferentially along the silicon (100) crystallographic orientation [20], the entire etching process is optimized for obtaining VA-SiNWs without the use of additional nanopatterning processes. The process condition for SiNW formation is set as the concentrations of HF, H_2_O_2_, and H_2_O in oxidizing hydrofluoric acid solution being 6.72, 0.79, and 48 M, respectively, initial silver thickness of 20 nm, and etchant temperature of 25°C. The control of silver thickness is crucial to the success of VA-SiNW formation since the morphology of thin silver film changes with the thickness [21]. The SiNW formation rate is about 1.2 μm/min. The control of wire length of SiNWs could be done by tuning the etching time. Residual silver catalysts are removed by nitric acid solution. In order to achieve the best alignment between SiNW and CNT patterns, a Fe/Al multilayer catalyst is deposited atop planar SiNWs by electron beam evaporation prior to the patterning process conducted by standard photolithography process using thick AZ-5214E PR to protect the catalysts and SiNWs at selective areas. The periodicity of the bundle array is controlled by the use of a photomask and at exposure/development conditions. Unmasked catalysts and SiNWs are then removed with hydrochloric acid and HNO_3_/HF/H_2_O etchants, respectively. The sample is then immersed in acetone for PR removal. Subsequent annealing is carried out at 750°C for 1 h at a pressure of about 0.1 Pa to form catalyst nanoparticles atop SiNW bundles. Thermal chemical vapor deposition (CVD) is then carried out with a growth temperature of 750°C and under a pressure of about 533 Pa maintained by flowing acetylene (C_2_H_2_) to grow CNT mats atop SiNW surface where the Fe/Al nanoparticles exist.

### Material and electrical characterization

All materials are characterized by using a field emission scanning electron microscope (SEM), an atomic force microscope (AFM), a Raman spectrometer, and a transmission electron microscope (TEM). The field emission characteristics and long-term stability tests of all samples are measured in a stainless chamber in which the samples serve as the cathode electrode. The current-voltage characteristic is determined by manipulating an electrometer under a chamber pressure of around 1.33 × 10^−5^ Pa. Accordingly, the current density to electric field characteristic of the sample is derived from the measured current-voltage curve, the inter-electrode distance (150 μm), and the sample area (10 × 10 mm^2^). The field emission performance is analyzed according to the Fowler-Nordheim (F-N) equation: *J* = *A*(*β*^2^*E*^2^/*Φ*)exp(−*BΦ*^3/2^/*βE*), where *J* is the field emission current density, *β* is the overall field enhancement factor, *E* is the applied electric field (V/μm), *A* (1.54 × 10^−6^ AeV/V^2^) and *B* (6.8 × 10^3^ eV^−3/2^ V/μm) are constants, and *Φ* is the work function (*Φ* is 4.8 and 4.15 eV for CNTs and SiNWs, respectively). The field enhancement factor is determined by the slope of the F-N plot.

For the electrical resistivity characterization of single SiNW, individual SiNWs are dispersed on the insulating Si_3_N_4_/n-Si template with pre-patterned Ti/Au microelectrodes. Electrical contacts of the two-terminal single-nanowire devices are fabricated by focused ion beam deposition using platinum as the metal electrode. Electrical measurements are carried out on an ultralow-current leakage cryogenic probe station. A semiconductor characterization system is utilized to source dc bias and measure current.

**Figure 2:**
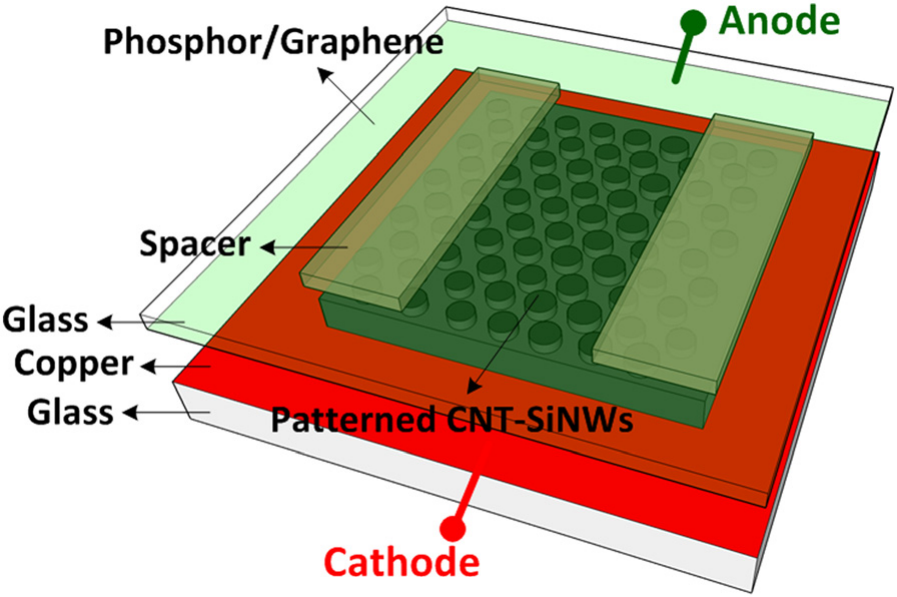
The schematic diagram of the fluorescent test setup.

Fluorescent tests are conducted to verify the uniformity of field emission from the CNT-SiNW heterojunction bundle array. In this experiment, the metal anode is replaced with a fluorescent screen in which the phosphor powder is uniformly smeared on the graphene electrode to collect emission electrons, as indicated in Figure 2. The inter-electrode distance is fixed by a 150-μm-thick glass spacer, and the chamber pressure is 6.6 × 10^−5^ Pa.

## Results and discussion

**Figure 3:**
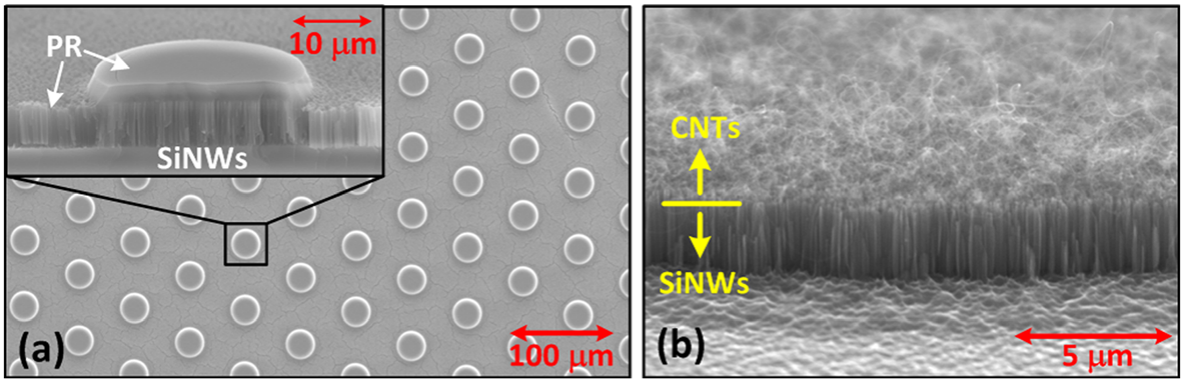
**SEM views of CNT-SiNW bundle arrays. (a)** Top and cross-sectional SEM image of SiNW bundle array protected by patterned PR template (after catalyst/SiNW removal step) and **(b)** cross-sectional SEM view of as-realized CNT-SiNW bundle array, indicating that CNTs are grown only atop SiNW bundles.

**Figure 4:**
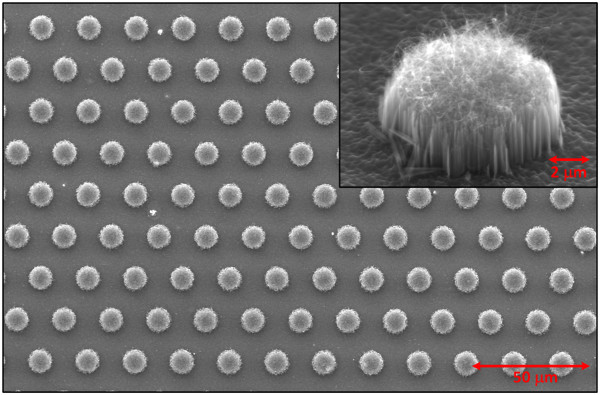
SEM image of CNT-SiNW bundle array with an inter-bundle distance of 20 μm.

CNT-SiNW heterojunctions take the benefits of superior thermal and electrical characteristics from CNTs. Though the electric field on the SiNWs is shielded by the CNTs such that the SiNWs may not be able to contribute to the emission current, the wire number density of as-grown CNTs atop SiNWs is found to be lower than the density of the underlying SiNWs which is at least 1 order of magnitude lower than the density of CNTs grown on flat silicon surface. The generated emission current from coarse CNTs as the emitters atop VA-SiNWs would be enhanced due to the release of electrostatic screening effect that typically limits the emission performance in dense CNTs. Nevertheless, the density of SiNWs is still high on the order of 10^9^ wires/cm^2^. The realization of an array of VA-SiNW bundles with a proper periodicity could further release the electrostatic screening effect and improve the field enhancement factor. This patterning approach had been verified by using an array of CNT bundles [13]. Previous report discovered that the emission strength in the edge of CNT bundles is 3.5 times higher than that in the center of the bundles [22]. The total edge area of the CNT-SiNW bundle array is then inversely proportional to the periodicity of the bundle array, indicating that smaller bundle periodicity would lead to better field emission performance. Prior to the patterning process shown in Figure 1, planar VA-SiNWs are prepared to have a nanowire length of around 2.44 μm, which is close to the optimal wire length of 2 μm according to the theoretical calculation taking both wire diameter and spacing into account. Figure 3a shows the SEM images of as-realized SiNW bundle array prior to the final PR removal step. The results indicate that thick PR could serve as the protecting mask to protect the SiNWs at selective areas from the chemical etching process, thus leads to the realization of a uniform SiNW bundle array. Figure 3b shows the cross-sectional SEM views of multi-walled CNT mats grown atop VA-SiNW bundle. The results verify the formation of CNTs only on top of SiNW bundles. According to the AFM results, the root mean square (rms) roughness of SiNW/catalyst removed areas is below 40 nm. The morphology of VA-SiNWs remains unchanged after the growth of CNTs. The bundle array of CNT-SiNW heterojunctions is fabricated with a hexagonal lattice and three periodicities (*P* = 20, 40, and 60 μm). Figure 4 shows the SEM image of the CNT-SiNW bundle array with an inter-bundle distance of 20 μm. The CNT mats cover the entire top surfaces of VA-SiNW bundles with precise alignment such that uniform bundle arrays of CNT-SiNW heterojunctions could be realized over a large sample area. We notice that the diameter of as-realized CNT-SiNW bundles shrinks by around 5 μm for all samples, which leads to a bundle diameter of 5, 15, and 25 μm for the corresponding CNT-SiNW bundle array with a periodicity of 20, 40, and 60 μm, respectively. The shrinkage may be due to the sinusoidal profile of the PR patterns such that the PR on the edge of the bundles cannot well protect the SiNWs during the wet chemical etching process.

**Figure 5:**
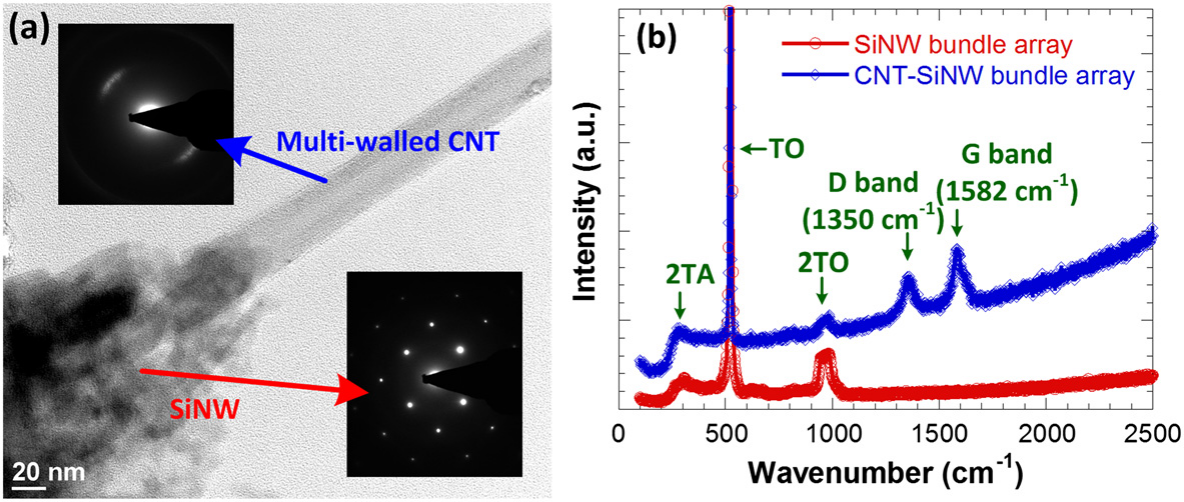
**Material characterization of CNT-SiNW heterojunction. (a)** TEM images of single CNT-SiNW heterojunction and the electron diffraction patterns of CNT and SiNW, respectively, and **(b)** Raman spectra comparisons of SiNW and CNT-SiNW bundle array. The curve for CNT-SiNW bundle array is offset in the plot for clarification.

Figure 5a shows the TEM image of individual CNT-SiNW heterojunction. As-grown CNTs are multi-walled with around 25 layers. The average diameters of as-realized SiNWs and CNTs are 90 and 30 nm, respectively. Their corresponding electron diffraction images reveal that SiNW retains the single-crystalline property whereas the CNT is polycrystalline. Raman spectra comparisons of SiNW and CNT-SiNW bundle array are shown in Figure 5b. The VA-SiNW bundle array shows characteristic Raman peaks at around 520, 960, and 300 cm^−1^, corresponding to the first-order transverse optical phonon mode (TO), the second-order transverse optical phonon mode (2TO), and the second-order transverse acoustic phonon mode (2TA), respectively [23]. The downshift and decreased intensity of the characteristic peak at 520 cm^−1^ for the cases of patterned CNT-SiNW heterojunctions indicate that there is a direct interaction between the SiNWs and CNTs [24]. After the patterned growth of CNTs, additional peaks appear in the Raman spectrum. The graphitic peak (G band) at 1,582 cm^−1^ is a peculiar characteristic of CNTs, which confirms that CNTs have grown on the surface of SiNWs [25]. The peak at 1,350 cm^−1^ (D band) indicates the defective layers on the surfaces of CVD-grown CNTs [26]. A relative determination of CNT quality can be found by comparing the ratio of the D band intensity (*I*_D_) over the G band intensity (*I*_G_). The *I*_D_/*I*_G_ ratio of multi-walled CNTs varies from 0.47 (with best quality) to 1.58 (highly defective) depending on the growth method and catalyst/barrier combinations [27]. The *I*_D_/*I*_G_ ratio of as-grown CNTs in this work is about 0.83, indicating a slightly higher defect density in CNTs. Nevertheless, a previous study found no clear correlation between CNT quality as determined by Raman spectroscopy and CNT field emission due to the effects CNT areal density has on field emission [27], indicating that it is electrostatic screening effect that dominates the performance of a field emitter. The cause of higher defect density in CNTs grown atop SiNWs and the influence of defect density in CNTs to the field emission performance of the CNT-SiNW bundle array require further investigations.

**Figure 6:**
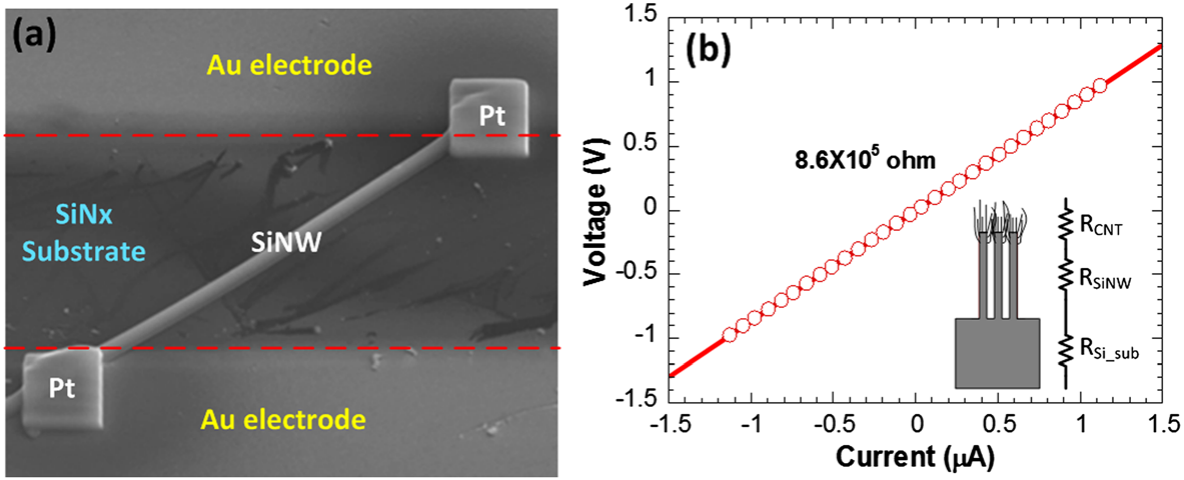
**Electrical characterization of single SiNW. (a)** Top SEM view of a two-terminal single-nanowire device and **(b)** its measured current-voltage characteristic at room temperature in air ambience.

For field emission applications, the use of CNTs as the emitter material would benefit from its metallic-like property and high thermal conductivity, while the advantages of using SiNWs would be the availability of mature and flexible processing technologies for silicon. Since the emission current flows through not only the CNTs but also the underlying SiNWs, the preservation of low bulk electrical resistivity in SiNWs is crucial to the success of this hybrid device. Figure 6a shows the top SEM view of a two-terminal single-nanowire device. This device is utilized to characterize the electrical resistivity of single SiNW. The diameter of the nanowire used in this experiment is 190 nm. The measured current-voltage characteristic of single SiNW is shown in Figure 6b. This experiment is conducted at room temperature in air ambience. The results reveal a linear relationship, indicating the ohmic contact condition of the nanowire device. Taking the nanowire length and diameter into account, the resistivity *R*_SiNW_ of single nanowire is estimated at around 2.14 Ω cm, which is close to the resistivity of original silicon substrate *R*_Si_sub_. Similar current-voltage characteristic and electrical resistivity is also obtained from a single-nanowire device with a wire diameter of 700 nm fabricated with the same process procedure. The results indicate that the electrical property of SiNWs comes from its bulk silicon material. The total electrical resistivity of individual CNT-SiNW heterojunction is mainly determined by the resistivity of SiNWs and silicon substrate, as indicated in the inset of Figure 6b. To further reduce the series resistance of the field emitter, heavily doped silicon wafer could be used as the starting substrate for SiNW formation.

**Figure 7:**
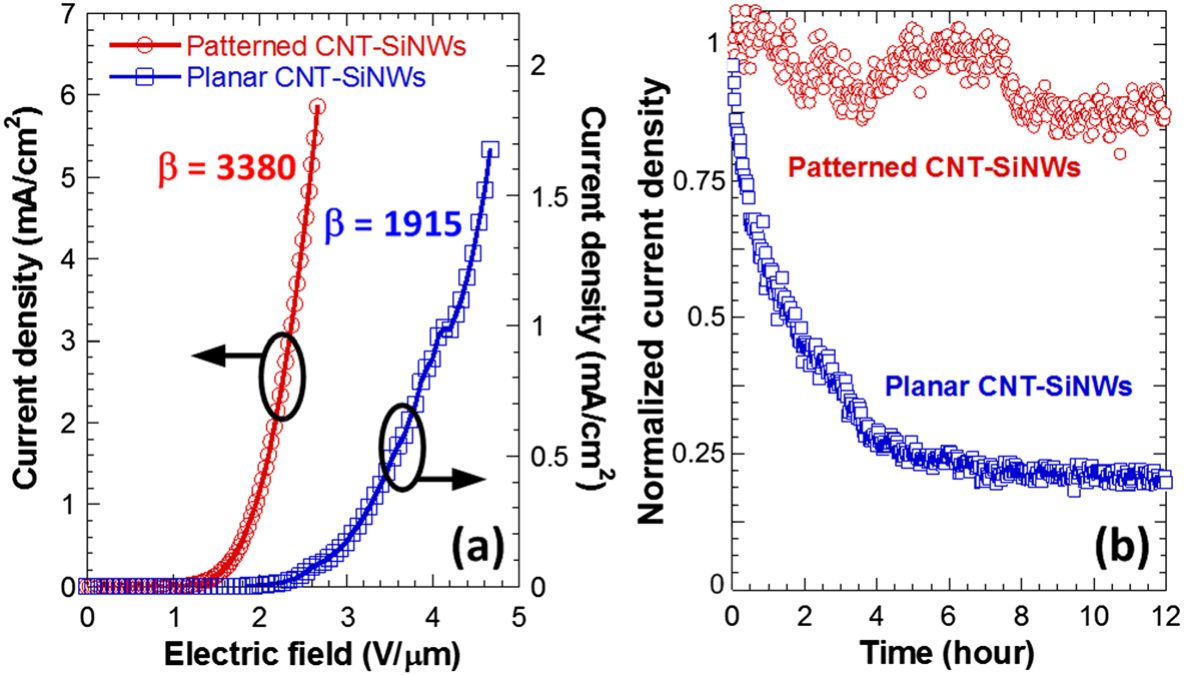
**Field emission characterization of CNT-SiNW field emitters. (a)** Comparison of electron field emission characteristics between patterned and planar CNT-SiNW field emitters and **(b)** long-term stability test of patterned and planar CNT-SiNW field emitters.

**Figure 8:**
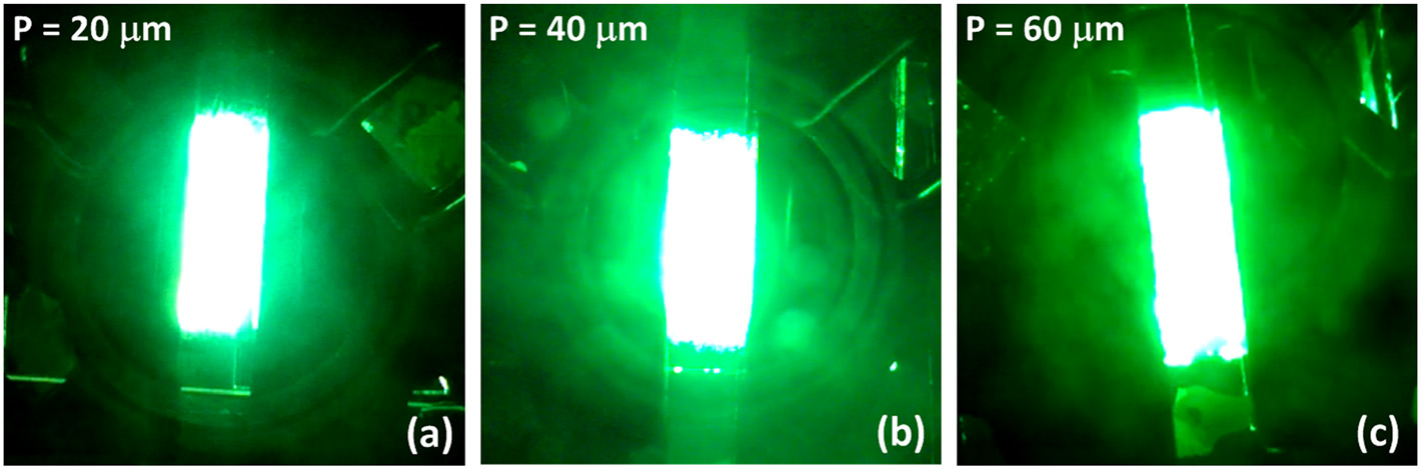
**Fluorescent screens of patterned CNT-SiNW field emitters.** The photographs of the fluorescent screens of patterned CNT-SiNW field emitters with an inter-bundle distance of **(a)** 20, **(b)** 40, and **(c)** 60 μm, respectively, during field emission measurements.

According to the measured electron field emission characteristics shown in Figure 7a, planar and patterned CNT-SiNW heterojunctions enable a turn-on electric field of 1.73 and 0.9 V/μm, respectively, while their emission current densities could reach 1.7 and 5.86 mA/cm^2^, respectively. Linear F-N plots derived from the measured field emission characteristics confirm that the emission detected originates from barrier tunneling quantum mechanical process. Therefore, the field enhancement factors of planar and patterned CNT-SiNW heterojunctions are calculated to be 1,915 and 3,380, respectively. Figure 7b shows the long-term field emission stability of planar and patterned CNT-SiNW heterojunctions. For comparison, different electric fields are applied to the respective devices in order to generate the same emission current density. Results indicate that the bundle array of CNT-SiNW heterojunctions exhibits relatively stable emission performance with a current fluctuation of about 10% over a 12-h period. Almost two times higher electric field is required for planar CNT-SiNW heterojunctions, thus leads to gradual emission degradation. The observed current-dependent degradation mechanism could be attributed to the interaction and energy dissipation during the solid-state transport of a high current density at the CNT apex, through the CNTs and SiNWs, or at the interface of CNT-SiNW and SiNW-substrate.Fluorescent tests are conducted to verify the uniformity of field emission from the CNT-SiNW bundle array. Figure 8 reveals the photographs of the fluorescent screens of the CNT-SiNW bundle array with an inter-bundle distance of 20, 40, and 60 μm, respectively, under an applied electric field of 2.66, 3, and 3.33 V/μm, respectively. Since a larger total edge area, thus better field emission performance, exists in the CNT-SiNW bundle array with a smaller periodicity, a higher electric field for samples with larger inter-bundle distances is applied in order to obtain similar emission current. Nevertheless, a uniform and bright fluorescent radiation is observed from all samples. The results indicate the existence of high-density and efficient field emitters on the proposed CNT-SiNW bundle arrays.

## Conclusions

The realization of field emitters based on arrayed CNT-SiNW heterojunctions would benefit from the superior thermal and electrical properties of CNTs while taking the advantages of flexible patterning technologies available for SiNWs. The proposed field emitter is capable of generating >5 mA/cm^2^ emission current density with a turn-on electric field of 0.9 V/μm. With the proposed fabrication strategy, it is possible to further scale down the periodicity of the CNT-SiNW bundle array while maintaining the verticality of the supporting structure. To investigate the electrical conductivity of as-realized SiNWs, a two-terminal single-nanowire device is fabricated and characterized. The results indicate similar electrical resistivity between individual SiNW and original bulk silicon substrate, meaning that no obvious electrical degradation after SiNW fabrication is observed. Therefore, further reducing the series resistance of the field emitters could be achieved by simply using low-resistivity silicon wafer as the starting substrate. Raman spectra of CNT-SiNW bundle arrays also reveal direct interactions between CNTs and SiNWs. The bundle array of CNT-SiNW heterojunctions exhibits relatively stable emission performance with a current fluctuation of about 10% over a 12-h period, as compared to planar CNT-SiNW counterparts. Finally, a bright and uniform radiation from the fluorescent screens of CNT-SiNW bundle arrays is observed regardless of its bundle periodicity. The results indicate the existence of high-density and efficient field emitters on the proposed CNT-SiNW bundle arrays.

## Competing interests

The authors declare that they have no competing interests.

## Authors' contributions

Y-JrH made substantial contributions to the conception, design, and implementation of the whole study. Y-JH contributed to the fluorescent test of CNT-SiNW field emitters. H-CC carried out the TEM characterization of CNT-SiNW heterjunction. K-YL and S-LL contributed to the data analysis as well as provided valuable advices to this study. All authors read and approved the final manuscript.

## Authors' information

Y-JrH is the assistant professor at the Department of Photonics, National Sun Yat-sen University, Kaohsiung, Taiwan. His research interests include the realization of silicon-based nanostructures for applications in various emerging fields. Y-JH and H-CC are graduate students at the Department of Electronic Engineering, National Taiwan University of Science and Technology, Taipei, Taiwan, under the supervision of professor Kuei-Yi Lee. Their researches focus on carbon nanotube- and graphene-based materials and devices. S-LL is the distinguished professor at the Department of Electronic Engineering, National Taiwan University of Science and Technology. His research interests include semiconductor optoelectronic devices and optical networking.
